# Affinity of Tannins to Cellulose: A Chromatographic Tool for Revealing Structure-Activity Patterns

**DOI:** 10.3390/molecules28145370

**Published:** 2023-07-13

**Authors:** Essi Suominen, Santeri Savila, Mimosa Sillanpää, Pia Damlin, Maarit Karonen

**Affiliations:** 1 Natural Chemistry Research Group, Department of Chemistry, University of Turku, FI-20014 Turku, Finland; essi.m.suominen@gmail.com (E.S.); savila.santeri@gmail.com (S.S.); mamsil@utu.fi (M.S.); 2 Materials Chemistry Research Group, Department of Chemistry, University of Turku, FI-20014 Turku, Finland; pia.damlin@utu.fi

**Keywords:** associations, binding, cell wall, hydrolysable tannin, insoluble dietary fibre, interactions, molecular weight, proanthocyanidin, UPLC-DAD

## Abstract

Food, feed and beverage processing brings tannins into contact with macromolecules, such as proteins and polysaccharides, leading to different chemical and physical interactions. The interactions of tannins with proteins are well known but less is known about the affinity of tannins to polysaccharides. We used bacterial cellulose from *nata de coco* as a model compound to investigate how tannins and cellulose interact by adsorption measurements using UPLC-DAD. We also explored how the structure of tannins influences these interactions. The model tannins included nine individual structurally different hydrolysable tannins (HTs) and eight well-defined proanthocyanidin (PA) fractions with different monomeric units, mean degree of polymerization and both A- and B-type linkages. Tannins were found to have both strong and weak interactions with bacterial cellulose, depending on the exact structure of the tannin. For HTs, the main structural features affecting the interactions were the structural flexibility of the HT molecule and the number of free galloyl groups. For PAs, prodelphinidins were found to have a higher affinity to cellulose than procyanidins. Similarly to HTs, the presence of free galloyl groups in galloylated PAs and the flexibility of the PA molecule led to a stronger interaction. Adsorption measurements by UPLC-DAD proved to be a sensitive and rapid tool to evaluate the affinity of tannins to cellulose.

## 1. Introduction

Tannins are widely distributed plant-specialized metabolites. They have many biological activities, including, for example, antioxidant [1,2,3,4,5,6], antimicrobial [7,8,9,10,11], antiviral [8,12,13,14], and anthelmintic or antiparasitic [15,16,17,18,19] activities. Tannins can also have important effects on both human and animal health and nutrition [2,20,21,22,23]. Tannins can be divided into three main groups: hydrolysable tannins (HTs), proanthocyanidins (PAs), and phlorotannins, which are mainly found only in marine organisms such as brown algae [24]. HTs include simple gallic acid derivatives, which contain five or less galloyl groups; gallotannins, which contain six or more galloyl groups of which at least one digalloyl group has been formed; and ellagitannins (ETs), which have either a hexahydroxydiphenoyl (HHDP) group or its modification(s) in the structure. PAs consist of two or more flavan-3-ol units and the two main classes of PAs are procyanidins (PCs) and prodelphinidins (PDs) consisting of (epi)catechin and (epi)gallocatechin units, respectively. Flavan-3-ol units are attached together via C4 → C8 or C4 → C6 bonds (B-type PAs) and there can also be one or more additional C2 → O → C7 or C2 → O → C5 ether bonds (A-type PAs). In addition, the hydroxyl groups in the PA structures can be substituted, for example, via galloylation or glycosylation.

In general, tannins are known to have interactions with biomacromolecules, such as proteins, carbohydrates and lipids. Of these, the complexation of tannins with proteins has been most intensively studied over the past fifty years [25], while less data are available about their interactions with lipids and polysaccharides [26,27,28,29]. In living intact plants and plant tissues, tannins, proteins and polysaccharides are separated but by tissue disruptions, for example, during food, feed or beverage processing or while chewing, these compounds can mix and react with each other [26,30]. Tannins can bind especially to the cell wall proteins and polysaccharides [26]. Previous studies have shown that apple cell walls have the capacity to bind apple PCs and these bindings can have consequences both on the organoleptic properties of food products, such as astringency and bitter taste, and the bioavailability of PCs [30,31]. The tannin–macromolecule interactions can affect the bioaccessibility, bioavailability and digestibility of tannins, and their physical, chemical and nutritional properties and bioactivities [27,32].

The interactions between tannins and polysaccharides can be studied by using cell wall fractions isolated from plants, for example, from apple [31,33,34,35] or grape [36], or model polysaccharides representing the different cell wall components, such as starch [35,37], pectin [35], xyloglucan [35], cellulose [35] or chitosan [38]. The interactions between tannins and polysaccharides can be covalent (irreversible) or non-covalent (reversible) [39]. The non-covalent interactions and complex formations have been more studied, due to their dietary applications [40,41]. These interactions are driven by hydrogen bonding or by hydrophobic interactions depending on the physicochemical properties and structural features of both the polyphenol and the polysaccharide studied, or on the environmental conditions used, such as temperature, pH and ionic strength [31,40,42,43]. For example, cyanidin-3-glucoside, ferulic acid and catechin showed, under various pH levels (3, 5 and 7), different adsorption to cellulose [43]. The adsorption of cyanidin-3-glucoside increased as pH increased from 3 to 5 and then decreased at pH 7, whereas the adsorption of ferulic acid increased little by little and the adsorption of catechin showed no change as the pH increased [43]. The different effect of cyanidin-3-glucoside may be explained by the influence of pH on the anthocyanin structures. Anthocyanidins exist in different forms depending on the pH of the solution: flavylium cations are dominant at very low pH, quinoidal forms are predominant at pH 2–4, chalcone and carbinol pseudoforms at pH 5–6 and when pH > 7, the anthocyanidins start to degrade, depending on their substituent groups [43,44]. The interactions of PCs and cell wall fractions were not affected by pH in the range of 2–7 [31], similar to the previous study where catechin adsorption was independent of pH [43], which meets expectations as tannins have a pK_a_ of around 9–10 [45,46].

**Figure 1:**
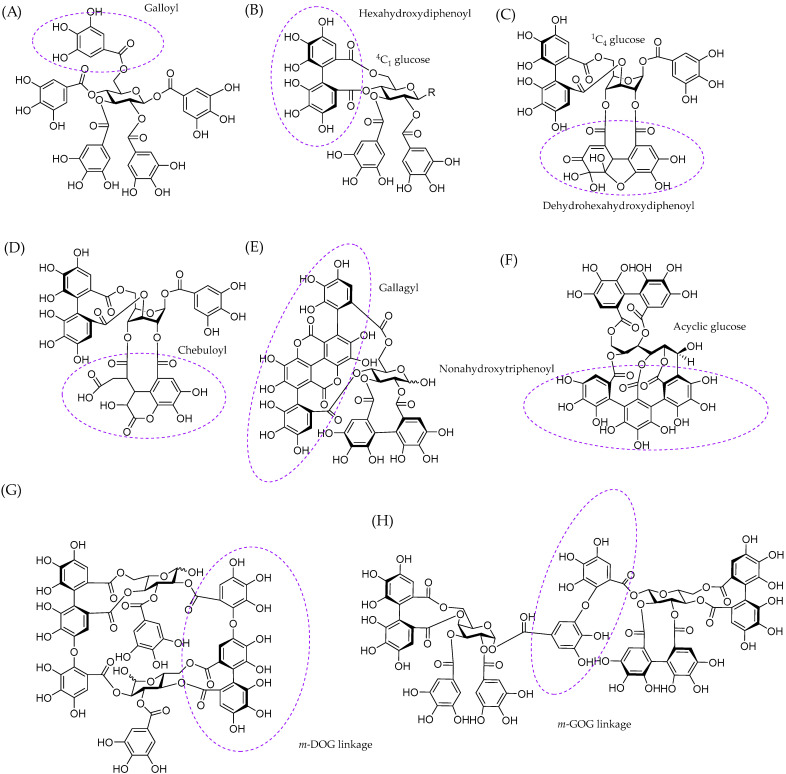
Hydrolysable tannins used in this study: (**A**) pentagalloylglucose, (**B**) R = OH = tellimagrandin I, R = galloyl = tellimagrandin II, (**C**) geraniin, (**D**) chebulagic acid, (**E**) punicalagin, (**F**) vescalagin, (**G**) oenothein B, and (**H**) gemin A. The different functional groups are highlighted with dotted circles. DOG refers to a linking unit where the hexahydroxydiphenoyl group is the *O*-donating polyphenolic unit and galloyl is the acceptor and GOG to a linking unit where both the *O*-donating and *O*-accepting units are galloyls.

**Figure 2:**
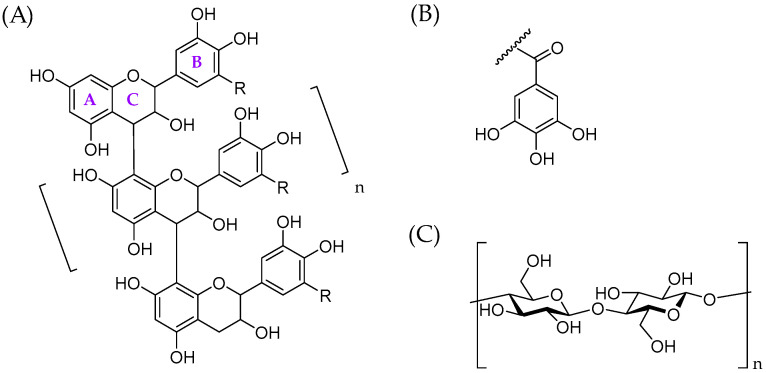
(**A**) A model structure for oligomeric B-type proanthocyanidins having C4 → C8 interflavanoid linkages. R = H, procyanidin, R = OH, prodelphinidin. Proanthocyanidins can also have C4 → C6 linkages or additional C2 → O → C7 or C2 → O → C5 ether bonds (A-type proanthocyanidins). The hydroxyl groups can also be galloylated, (**B**) galloyl group, and (**C**) generic structure for cellulose.

Previous studies have used chromatographic supports, such as cellulose or modified dextrans, to evaluate the affinity of tannins to polysaccharides, as discussed by Le Bourvellec and Renard [26]. In this study, we selected bacterial cellulose as a model polysaccharide. Bacterial cellulose is a bacteria-produced polysaccharide with a nanofibrous structure: it has a high purity and crystallinity and it is structurally identical to plant-derived cellulose, i.e., having a relatively hydrophobic nature in comparison to other cell wall polysaccharides and a rigid form of fibrils facilitating the binding via reducing entropic barriers [47,48,49]. Previously, bacterial cellulose from *Gluconacetobacter xylinus* has been used to study the binding of dietary phenolic compounds to cellulose [48]. However, we decided to use bacterial cellulose obtained from *nata de coco* where it is produced during coconut water fermentation by the bacteria *Acetobacter xylinum* [50] and, therefore, no cultivation is needed. The bacterial cellulose obtained in this way possesses excellent physicochemical and mechanical properties, as described in detail by Popa et al. [49]; furthermore it is also ecological, biocompatible, and biodegradable. The main aim of this study was to investigate the interactions and adsorption behaviour of different tannins with bacterial cellulose using well-defined tannin structures and simple adsorption measurements by UPLC-DAD. These tannin structures included nine individual HTs (Figure 1), i.e., eight different ETs and pentagalloylglucose, and eight well-characterized PA fractions (Figure 2). The main objectives were to show that tannins have the capacity to bind to cellulose, understand the interactions between tannins and cellulose in detail, and reveal the structural features of tannins affecting these interactions.

## 2. Results and Discussion

### 2.1. Characterization of Model Tannins

**Figure 3:**
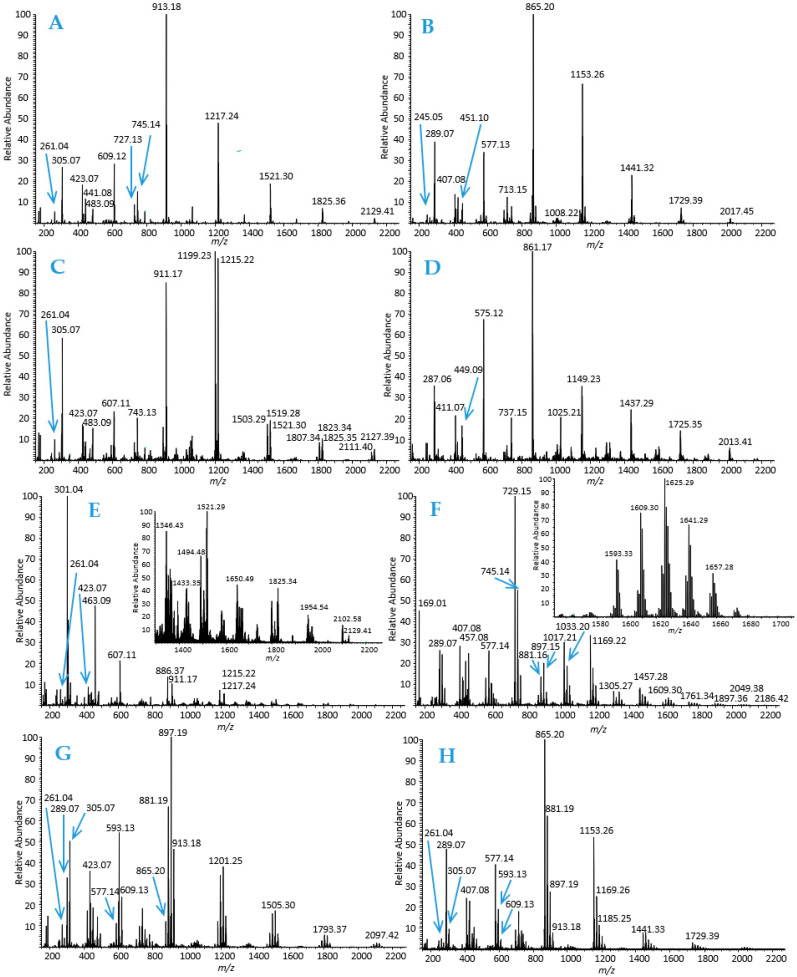
Total mass spectra of proanthocyanidins (PAs) in the purified PA fractions: (**A**) B-type prodelphinidins (PDs), (**B**) B-type procyanidins (PCs), (**C**) A-type PDs, (**D**) A-type PCs, (**E**) PDs with a higher mean degree of polymerization than in aforementioned B- and A-type PDs, (**F**) galloylated PAs containing both PC and PD units (PC/PD mixture), (**G**) PD-rich PC/PD mixture, and (**H**) PC-rich PC/PD mixture.

The interactions and adsorption behaviour of individual HTs and well-characterized PA fractions with bacterial cellulose were studied by simple adsorption measurements using UPLC-DAD. Model HTs were isolated, purified and characterized as previously reported; for further structural information and spectroscopic data of model HTs, see the supplementary material in Virtanen et al. [51]. The model PAs were isolated by the semipreparative liquid chromatographic technique [52] which allowed the production of chemically and chromatographically well-resolved PA fractions with divergent subunit composition and mean degree of polymerization (mDP). The obtained novel eight PA fractions included: (1) B-type PDs, (2) B-type PCs, (3) A-type PDs, (4) A-type PCs, (5) PDs with higher mDP, (6) galloylated PAs containing both PC and PD units (PC/PD mixture), (7) PD-rich PC/PD mixture, and (8) PC-rich PC/PD mixture (Figure 3, Tables S1–S8). PAs in these fractions were characterized by high-resolution UPLC-MS/MS based on their singly and multiply charged molecular ions and characteristic fragmentation patterns, i.e., retro-Diels–Alder (RDA) fragmentation, quinone-methide (QM) cleavage and heterocyclic ring fission (HRF), as previously discussed in detail in Karonen et al. ([53] and the references therein).

Based on the overall appearance of the total mass spectra of PA fractions, it is evident that the PA compositions are very different. The mass spectrometric data of B-type PDs (Figure 3A) show a series of [M-H]^−^ ions corresponding for pure PD oligomers starting from a PD dimer exhibiting a singly charged ion at *m*/*z* 609.12 and continuing by the ions at *m*/*z* 913.18 (PD trimers), 1217.24 (PD tetramers), 1521.30 (PD pentamers) and accordingly with a mass difference of 304.06 Da for singly charged ions corresponding to one (epi)gallocatechin unit attached via one C-C bond. The mass spectrum exhibits the characteristic fragment ions at *m*/*z* 261 (from the monomeric unit), 303 and 305 (QM), 423 and 441 (RDA) and 467 (HRF). In addition, a series of [M-2H]^2−^ ions separated by 152.03 Da starting from *m*/*z* 1064 (PD heptamer) can be detected. Similarly, the mass spectrometric data of B-type PCs (Figure 3B) show a series of [M-H]^−^ ions corresponding for pure PC oligomers starting from PC dimers exhibiting singly charged ions at *m*/*z* 577.13 and continuing by the ions + 288.06 Da for singly charged ions, i.e., PC trimers at *m*/*z* 865.20, PC tetramers at *m*/*z* 1153.26, PC pentamers at *m*/*z* 1441.32 and so forth. The mass spectrum also exhibits the characteristic fragment ions at *m*/*z* 245 (from monomeric unit), 287 and 289 (QM), 407 and 425 (RDA) and 451 (HRF) [53]. In addition, a series of [M-2H]^2−^ ions separated by 144.03 Da starting from *m*/*z* 1008 (PC heptamer) can be detected.

A-type PDs (Figure 3C) exhibited similar ions to B-type PDs but with a mass difference of 2 Da corresponding to the presence of the additional ether bond in the structure. For example, the A-type PD dimer exhibited an [M-H]^−^ ion at *m*/*z* 607.11 and the PD trimer at *m*/*z* 911.07 (Figure 3C) in comparison to B-type PD dimer and trimer having the corresponding molecular ions at *m*/*z* 609.12 and 913.18, respectively (Figure 3A). In addition, a mass difference of 16 Da was observed, hinting that some of the PD oligomers contained one PC unit in addition to PD units. Similarly, A-type PCs (Figure 3D) exhibited ions with a mass difference of 2 Da in comparison to the ions of B-type PCs. The A-type PC dimer produced the [M-H]^−^ ion at *m*/*z* 575.12 and the trimer at *m*/*z* 861.17 indicating that there were two additional ether bonds in the structure. The same mass difference of 2 × 2 Da = 4 Da and thereby the existence of two ether bonds was observed also for other oligomeric A-type PCs (Figure 3D).

In addition to smaller B-type and A-type PDs (in Figure 3A,C), we wanted to use PDs with a higher mDP as a model (Figure 3E). This PD fraction contained both A- and B-type PDs, see for example the [M-H]^−^ ions at *m*/*z* 1215 and 1217 corresponding to A-type and B-type PD tetramers, respectively. The mass spectrum of this fraction is already quite complex and has many isotopic patterns, indicating the presence of different PA oligomers and their isomers (Figure 3E). Higher PDs also contained traces of other polyphenols. At this point, it must be remembered that the efficiency of ionization decreases when the degree of polymerization of PAs increases and the ionization of other phenolic compounds is more intensive [53]. Therefore, the ions at *m*/*z* 301 and 463, corresponding to impurities, seem to be very intensive, though they are minor components in the fractions based on UV absorbance.

One of the model PA fractions contained galloylated PAs consisting of both PC and PD units (PC/PD mixture), as shown in Figure 3F. The presence of galloylation was evidenced by the mass difference of 152 Da, see for example a PC dimer at *m*/*z* 577 and a galloylated PC dimer at *m*/*z* 729. Some of the PAs contained two galloyl groups, 2 × 152 Da = 304 Da, in which case the mass difference with integer precision also agreed with an additional PD unit. However, using an ultrahigh-resolution mass spectrometer, we can differentiate these two as the exact mass of one galloyl group (C_7_H_4_O_4_) is 152.01096 Da and of two galloyls is 304.0219 Da, while the exact mass of (epi)gallocatechin as an extension unit is 304.05831 Da. This can be seen, for example, in the case of the [M-H]^−^ ion at *m*/*z* 881; the exact mass measured 881.15791 Da corresponds to a galloylated PC dimer having two galloyl groups (C_44_H_34_O_20_, calculated 882.16435) not to a trimeric PA consisting of two PC and one PD units (C_45_H_38_O_19_, calculated 882.20074). The characteristic fragments ions for galloylated PAs were detected at *m*/*z* 169 (gallic acid); 287, 305, 441, and 457 (QM); 407, 423, 425 and 441 (RDA); 577 and 593 (non-galloylated dimers); 603 and 619 (HRF, minor). These fragments are consistent with the previous data presented in Karonen et al. ([53] and its supplementary material).

Two PA fractions containing PC/PD mixtures where also used: one fraction with more PD units in the PA structures (PD-rich PC/PD mixture in Figure 3G) and the other one with more PC units in the PA structures (PC-rich PC/PD mixture in Figure 3H). The structural differences can be nicely seen by comparing the [M-H]^−^ ions of different PA trimers in these fractions. In the PD-rich PC/PD mixture (Figure 3G), the trimers exhibit [M-H]^−^ ions at *m*/*z* 865 (PC-PC-PC, minor ion), 881 (PC-PC-PD, the second most intensive ion), 897 (PC-PD-PD, the most intensive ion) and 913 (PD-PD-PD, smaller ion). In the PC-rich PC/PD mixture (Figure 3H), the trimers exhibit [M-H]^−^ ions at *m*/*z* 865 (PC-PC-PC, the most intensive ion), 881 (PC-PC-PD, the second most intensive ion), 897 (PC-PD-PD, smaller ion) and 913 (PD-PD-PD, minor ion). In these trimers, the marking “PC-PC-PD” means that the PA trimer contains two PC units and one PD unit but the sequential order is not precisely determined; it can also be PC-PD-PC or PD-PC-PC.

### 2.2. Characterization of Bacterial Cellulose

**Figure 4:**
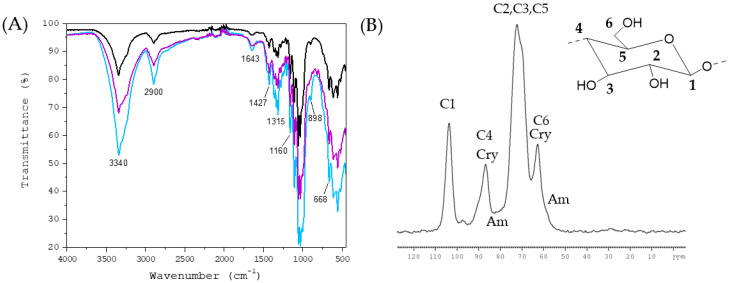
Characterization of the bacterial cellulose prepared from *nata de coco* according to the method of Veliz et al. [59]: (**A**) FTIR spectra of three different batches (colored lines) of bacterial cellulose and (**B**) NMR spectrum of one sample of bacterial cellulose with an illustrative cellulose structure with assigned carbons (C1–C6). Cry refers to crystalline and Am to amorphous domains of cellulose.

The bacterial cellulose was characterized by Attenuated Total Reflection–Fourier Transform InfraRed spectroscopy (ATR-FTIR) and ^13^C cross-polarization magic angle spinning nuclear magnetic resonance (^13^C-CP-MAS-NMR) spectroscopy. FTIR spectra of three batches of model bacterial cellulose prepared from *nata de coco*, confirming both the reproducibility of preparation and the structure and purity of the bacterial cellulose produced, are presented in Figure 4A. The FTIR spectrum of a sample is commonly denoted as a fingerprint as it provides a unique spectral signature of the sample in question. It is important to notice that unless fingerprint peaks can confirm a structure such as that of cellulose, the shape of peaks may vary, depending on the origin of cellulose. In the FTIR spectra, the band around 3340 cm^−1^ and the shoulder at 3234 cm^−1^ are assigned to O-H stretching and the peak around 2900 cm^−1^ to C-H symmetric stretching. The H-O-H deformation due to adsorbed water can be observed at 1643 cm^−1^. The band at 1160 cm^−1^ is attributed to the C-O-C and the peaks at 1000–1060 cm^−1^ to C-O-stretching vibrations, respectively. The band at 898 cm^−1^ is characteristic of β-glycosidic linkage between glucose units. In addition, the peaks at about 1315 cm^−1^ indicate C-H bending and about 1427 cm^−1^ CH_2_ bending. The peak at 668 cm^−1^ corresponds to the out-of-plane bending of COH. The spectra obtained were similar as previously presented for bacterial cellulose prepared from *nata de coco* by Halib et al. [54] and showed the distinguished peaks reported for pure cellulose [55], thus confirming the purity of the cellulose used in this study. The ^13^C CP-MAS-NMR spectrum of bacterial cellulose (Figure 4B) was identical with the literature [56,57], having distinct signals at about 104 ppm (C1), 87 ppm (C4), in the 70–80 range (C2, C3 and C5) and 63 ppm (C6) attributing to the crystalline (ordered) domains of cellulose and more ambiguous signals at 83 ppm (C4) and 60 ppm (C6) corresponding to the amorphous (disordered) regions of cellulose. The adsorption can occur both on the crystalline and amorphous domains of cellulose: low concentration in the crystalline regions is preferred, but when the concentration of polyphenol is high enough, adsorption can also take place in amorphous domains [26,58].

### 2.3. Interactions between Tannins and Bacterial Cellulose

**Figure 5:**
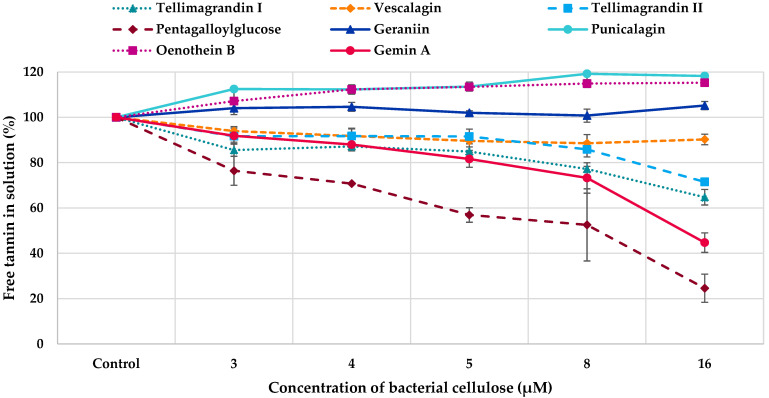
Amounts of free hydrolysable tannins (HTs) in solution (%) in comparison to control HT solution after the incubation with bacterial cellulose.

The affinity of tannins to bacterial cellulose was evaluated using simple adsorption measurements in which the tannins were incubated in solutions with different concentrations of bacterial cellulose and after the incubation, the amount of free (unbound) tannins in the solution was measured through absorbance spectroscopy by UPLC-DAD and compared to the amount of tannins in the control solution (no interaction with the cellulose). Different concentrations of bacterial cellulose were used as the relative concentrations of both tannin and polysaccharide affect the interactions [26]. We used UPLC-DAD at 280 nm for the measurements, but the experiments can also be performed with simpler instruments, such as HPLC-UV, as the chromatographic performance does not need to be that high since pure tannins or tannin fractions are studied and only one wavelength is needed. The affinities of HTs to bacterial cellulose are presented in Figure 5. The strongest interaction among HTs was detected for pentagalloylglucose. Other active HTs were tellimagrandins I and II, gemin A and vescalagin. The rest of the ETs did not interact with bacterial cellulose. In general, the replicate measurements were repeatable but more variation was detected for pentagalloylglucose, which might be due to its hydrophobicity. In general, the binding of HTs to bacterial cellulose improved when the concentration of bacterial cellulose increased within the concentration range used.

**Figure 6:**
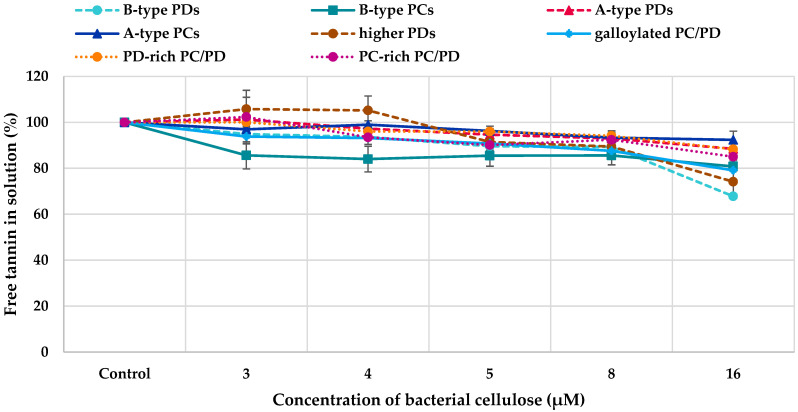
Amounts of free proanthocyanidins (PAs) in solution (%) in comparison to control PA solution after incubation with bacterial cellulose. PC refers to procyanidins, PC/PD to a PA mixture containing both PC and PD units in the PA structures, and PD to prodelphinidins.

All PA fractions interacted with bacterial cellulose (Figure 6). However, the differences between different PA fractions were small. In general, the binding of PAs to bacterial cellulose increased with the increase in the concentration of bacterial cellulose within the concentration range used, similarly to HTs. The only exception was the fraction containing B-type PCs: the amount of free B-type PCs was almost the same in different molar ratios of tannin to bacterial cellulose. In general, the interactions were stronger for HTs as a smaller concentration was adequate for the interactions between HTs and bacterial cellulose (0.03 mM) than for the interactions between PAs and bacterial cellulose (0.06 mM). Further, all HTs did not interact with bacterial cellulose (Figure 5), whereas all PA fractions had some affinity to cellulose (Figure 6).

### 2.4. Structure-Activity Patterns

The diverse set of tannins allowed us to evaluate the effects of different structural features on the interactions between tannins and bacterial cellulose. For the detailed inspection, we used the data obtained by highest concentration of cellulose used. The interactions of HTs with bacterial cellulose together with their main structural features and functional groups affecting these interactions are presented in Table 1.

The interaction was the strongest, i.e., the amount of free (unbound) tannin in the solution was the smallest, for pentagalloylglucose which is a hydrophobic, spherical, and flexible HT with five free galloyl groups. The second-largest effect was detected for gemin A, which is a flexible dimer with three HHDP and two free galloyl groups (Figure 1H) and the monomeric units are attached together via *m*-GOG linkage, where both the *O*-donating and *O*-accepting units are galloyls [60]. The interactions were also strong for tellimagrandins I and II, having both one HHDP group and two and three free galloyls, respectively. The effect of HHDP group on the HT–cellulose interactions can be evaluated by comparing the binding of tellimagrandin II and pentagalloylglucose, which are structurally very similar. The only difference is that tellimagrandin II has an (*S*)-HHDP group in O4~O6 of glucose (Figure 1B) while pentagalloylglucose has instead two free galloyls (Figure 1A). The affinity of tellimagrandin II to cellulose was weaker than that of pentagalloylglucose (Table 1) showing that the presence of the HHDP group decreases the affinity to cellulose. Previous results, obtained by thin-layer chromatographic studies on cellulose, have shown that ETs have weaker interactions with cellulose than gallotannins that have similar molecular weights and the same numbers of galloyl and OH groups [61]. This is most probably related to the hydrophobicities of these tannins [62] and the reduced flexibility of galloyl groups due to the formation of the HHDP group. However, it must be noted that the affinity of tellimagrandin II to cellulose was not low, even though it was lower than that of pentagalloylglucose. It should also be noted that the affinity of gemin A to cellulose was stronger in comparison to its monomeric unit, tellimagrandin II. This finding is in line with common knowledge that HTs with a higher molecular weight bind strongly to macromolecules [28,39,61,63,64]. In addition to these HTs with a glucopyranose core, the acyclic ellagitannin, vescalagin, showed some affinity to cellulose. This was surprising as the structure of vescalagin is rather rigid because of the nonahydroxytriphenoyl (NHTP) group at C1~O2~O3~O5 of glucose and the (*S*)-HHDP group at O4~O6 (Figure 1). In addition, vescalagin is highly hydrophilic [62]. Previously, vescalagin has shown weak binding to Sephadex G-25 and G-50 dextran gels modelling the polysaccharide matrix [39]. Also in this research, the weak binding was thought to be related to the rigidity of vescalagin structure.

The rest of the HTs tested, geraniin, chebulagic acid, punicalagin and oenothein B, did not interact with bacterial cellulose within the concentrations and conditions used. One reason for this might be the different functional groups in the structure, i.e., a dehydro-HHDP group in geraniin, a chebuloyl group in chebulagic acid and a gallagyl group in punicalagin. This might also be linked to the conformation of the glucopyranose core. In pentagalloylglucose, tellimagrandins I and II, and gemin A, the glucopyranose is in ^4^C_1_ conformation whereas it is in thermodynamically less favourable ^1^C_4_ conformation in geraniin and chebulagic acid (Figure 1). Similar observation has been made previously: ETs having a ^4^C_1_-D-glucopyranose conformation bound more strongly to Sephadex materials than the ones with ^1^C_4_ conformation [39]. The third reason can be the rigidity of the structure. Oenothein B is a rigid macrocyclic dimer where the monomeric units are attached together by two *m*-DOG-type linkages, whereas flexible dimer gemin A has only one *m*-GOG linkage.

In addition, the solubility of these inactive ETs seemed to increase in the presence of bacterial cellulose as their amounts in the solutions were higher after the incubation with cellulose (Figure 5). However, all tannin solutions were initially made so that the tannins were first dissolved in a few droplets of ethanol in order to make them all fully dissolved and then diluted into final concentration with water. In general, this phenomenon might be linked to the self-association of HT molecules [65]. Most probably, the presence of bacterial cellulose can disturb this self-association, resulting in the higher absorbance in UV detection. In the case of active HTs, the affinity to cellulose was so high that the effect of self-association did not disturb the measurements, whereas in the case inactive HTs, the changes in the self-association yielded higher absorbance. The solubility of chebulagic acid almost doubled and it had no interaction with the bacterial cellulose; therefore, it is not shown in Figure 5.

Altogether, our results obtained by diverse HTs highlighted the role of free galloyl groups in the HT structures in the HT–bacterial cellulose interactions in addition to the flexibility of HT structures. Previous results, obtained by thin-layer chromatographic studies on cellulose, have shown that the affinity of HTs to cellulose correlates with the number and flexibility of galloyl groups and the hydrophobicity of HTs [61]. However, the affinity of tellimagrandin I to cellulose was stronger than that of tellimagrandin II, even though tellimagrandin I has only two free galloyls whereas tellimagrandin II has three. The stronger interaction of tellimagrandin I might be explained by a free –OH group at C-1 of glucose in tellimagrandin I. 

As mentioned above, all PA fractions interacted with bacterial cellulose. For the evaluation of different structural features of PAs on their affinity to cellulose, we used the detailed PA composition (Figure 3) and the mDPs and PC:PD ratios of PAs and the amount of free PA in solution after incubation with 16 µM cellulose (Table 2). The strength of the interaction was the strongest for B-type PDs. The comparison of the affinity of pure B-type PDs to that of pure B-type PCs shows that B-type PDs have higher affinity than B-type PCs, i.e., the amount of free PDs in solution is smaller after the incubation than the amount of free PCs (Table 2). The same is true for A-type PDs and PCs: A-type PDs have slightly higher affinity to cellulose than A-type PCs. No corresponding comparison of the effects of PDs and PC can be found in the literature. There is only one study with grape skin and seed PAs, where the relative proportions of PDs and PDs have been compared [66]. Nevertheless, in this study the PA fractions were complex and other variable factors (mDP and galloylation %) were present and, therefore, the role of the hydroxylation pattern could not be concluded [28,66]. However, this pattern, obtained for pure PDs and PCs, was not evident for the PC/PD mixtures. The PD-rich PC/PD mixture having approximately 79% of PD units in its structure had a about the same affinity to the cellulose as the PC-rich PC/PD mixture having approximately 77% of PC units in its structure, i.e., 88% and 85% of PAs were free in the solution, respectively. One reason for this could be the sequential order of monomeric units. Based on the data, we cannot reliably conclude whether the terminal units in the PA structures are PDs or PCs. Another reason could be the stereochemistry of monomeric flavan-3-ol units. For example, the presence of (+)-catechin in the extension units makes the PA conformation more open and flexible, enabling the formation of hydrogen bonds and hydrophobic interactions [28,31].

Based on the literature search and the review of Le Bourvellec and Renald [28], there are no studies on the interactions between A-type PAs and polysaccharides or cell wall materials. However, the decrease in cranberry A-type PCs during blanching has been suspected to be caused by the binding of these PCs to the cell wall material [28,68]. Our results clearly show that A-type PAs have interactions with cellulose (Table 2) and that the interactions seem to be slightly stronger for A-type PDs than for A-type PCs. A-type PDs and PCs had similar mDPs (11.0 and 10.4, respectively) and, therefore, the difference in their affinities to cellulose can be concluded to be related to their constitutive units. The interactions of A-type PAs with bacterial cellulose were weaker in comparison to B-type PAs (A-type PDs < B-type PDs and A-type PCs < B-type PCs, Table 2). A-type PAs have more rigid structures in comparison to B-type PAs because of the additional ether bond(s). This means that the structural flexibility also played a role in PA–bacterial cellulose interactions.

The galloylation of PAs increased their affinity to cellulose. The galloylated PC/PD mixture was structurally very similar to PC-rich PC/PD mixture in regard to mDP (4.6 and 6.2, respectively) and PC% (72 and 77, respectively) but had stronger interactions with cellulose, which must therefore be due to the galloylation (Table 2). This observation is supported by the literature. Previously, higher interactions with apple cell wall materials were observed with the increased degree of galloylation of apple PCs [31,34]. Correspondingly, galloylation also increased the interaction of catechins with oat *β*-glucan [41]. The increased affinity due to galloylation can be a result of the increased number of binding sites, increased hydrophobic character or greater flexibility [28].

Based on the literature, we expected the interaction to be higher with the increased mDP of PAs. For example, for apple PCs, higher interaction has been detected with an increased DP or higher molecular weight [31,34] and the interactions between commercial PAs and grape skin cell wall material have shown that the affinity of cell walls for PAs increases with the increasing molecular mass of PAs and is more related with the PA molecular mass than with the percentage of galloylation of PAs [69]. In general, the interactions between PAs and macromolecules, either proteins or polysaccharides, have been associated both with the presence of hydroxyl groups and aromatic rings forming hydrogen bonds and hydrophobic interactions and the molecular size, meaning that larger PAs have more binding sites than the shorter ones [28,39]. In our study, the effect of the size of the molecule was not that evident and our series of PA fractions did not allow us to compare the role of mDP as a single factor. The highest mDPs of PAs were in the fractions also having rigid A-type PAs, so the effect of increased mDP might be masked with the rigidity of PAs, for example, in the case of higher PDs. If we compare the pure B-type PAs, PDs with an mDP of 10.4 had stronger interactions with cellulose than PCs with an mDP of 5.3, but this could also be explained by the generally higher interactions of PDs.

Previous studies have emphasized the role of stereochemistry, conformational flexibility, and molecular size, especially in the case of cell wall materials as a model [26]. Our results highlighted the flexibility too, but also indicated that, in general, PDs might have more ability to bind to bacterial cellulose. Previously, the physicochemical features of tannins have been generalized so that pentagalloylglucose and gallotannins are hydrophobic and flexible, whereas ETs are hydrophilic, more spherical, propeller-like and rigid, and PAs are hydrophilic, elongated, threadlike and flexible—all of these being important parameters in tannin–protein interactions [70]. This is of course a generalization and does not take into account individual structural factors, such as the stiffening effect of the A-type bond in PAs compared to the B-type PA structure. Therefore, instead of classification and tannin groups, individual structures need to be studied. However, in the big picture this generalization explains well the results obtained in this study and the observed similarities and differences between different tannin classes and their interactions with cellulose. Generally speaking, the mechanism for the interactions between polyphenols and polysaccharides is thought to be the same than for the interactions between polyphenols and proteins, i.e., the adsorption mediated by the hydrogen bonding and hydrophobic interactions [26,39].

## 3. Materials and Methods

### 3.1. Model Tannins

The model tannins included both HTs and PAs. The HTs used were tellimagrandin I, vescalagin, tellimagrandin II, pentagalloylglucose, geraniin, chebulagic acid, punicalagin, gemin A, and oenothein B. HTs were selected so that they represented different structural features and biosynthetic pathways, as described in Table 1. Ellagitannins were extracted, isolated, and purified following the previously described methods utilizing extraction with aqueous acetone, column chromatography on Sephadex LH-20 and preparative and semipreparative HPLC [64,71,72,73,74,75,76]. Tellimagrandins I and II were obtained from *Filipendula ulmaria* flowers, vescalagin from *Quercus robur* acorns, geraniin from *Geranium sylvaticum* leaves, chebulagic acid and punicalagin from *Terminalia chebula* (commercial leaf power purchased from Banyan Botanicals, Albuquerque, NM), gemin A from *Geum urbanum* leaves and oenothein B from *Epilobium angustifolium* flowers. Pentagalloylglucose was prepared from tannic acid purchased from J.T. Baker (Denventer, The Netherlands), as described in Salminen et al. (2001). The structures of HTs were confirmed by UPLC-DAD-ESI-MS/MS and NMR spectroscopic techniques similarly to our previous work [18,51,64]. Eight well-defined PA fractions were obtained according the semipreparative HPLC method outlined by Leppä et al. [52]. B-type PDs were isolated from *Acacia karroo* leaves, B-type PCs from *Heritiera solomonensis* leaves, A-type PDs from *Pellaea rotundifolia*, A-type PCs from *Ixora coccinea*, higher PDs from *Acacia karroo* leaves, galloylated PC/PD mixture *Coccoloba uvifera* leaves, PD-rich PC/PD mixture from *Podocarpus macrophyllus* leaves and PC-rich PC/PD mixture from *Ceplahotaxus harringtonia* subsp. *drupacea* leaves. The selection of appropriate PA-rich plant species was made based on the previous screening of 300 plant samples [53,77]. The obtained PA fractions were characterized both by ultrahigh-resolution UPLC-DAD-ESI-Qorbitrap-MS/MS [53] and multiple reaction-monitoring methods by UPLC-DAD-ESI-QQQ-MS/MS [67]. The mass spectrometric data of PAs are presented in Figure 3 and the main ions listed in Tables S1–S8 in Supplementary Materials.

### 3.2. Bacterial Cellulose

The bacterial cellulose was prepared according to the method of Veliz et al. [59]. In this method, bacterial cellulose is extracted from *nata de coco* where it has been formed by coconut water fermentation using the bacteria *Acetobacter xylinum* [50]. *Nata de coco* (BC 5220 Buenas Nata White, Philippines) was obtained from a local grocery store. Previous studies have shown that *nata de coco* manufactured for food use is a reliable source of bacterial cellulose [54]. The sweet jelly-like cubes were first washed with ultrapure MilliQ water and then they were crushed by mixing them in a blender with ultrapure water. The obtained mixture was filtered through a Buchner funnel using a Whatman filter paper (541, CAT No. 1541-110), after which the filtrate residue was purified by heating it at 80 °C in 0.1 M NaOH solution for 20 min [59]. After that, the bacterial cellulose mixture was filtered again through a Buchner funnel and washed with ultrapure water until the pH of the water passing through was neutral. Finally, the purified bacterial cellulose was freeze-dried.

The freeze-dried bacterial cellulose samples were characterized by Fourier Transform InfraRed (FTIR) spectroscopy using a Bruker VERTEX 70 FTIR spectrometer (Bruker Optic GmbH, Ettlingen, Germany). The spectrometer was equipped with a Harrick VideoMVP™ diamond ATR (attenuated total reflection) accessory (Pleasantville, NY, USA) and a room temperature operated deuterated triglycine sulfate (DTGS) detector. For each spectrum of the internally reflected IR beam, a total of 128 scans were measured between 4000 cm^−1^ and 450 cm^−1^ at 4 cm^−1^ spectral resolution. A clean diamond crystal surface was used as the background in all measurements. In addition, ^13^C solid state NMR spectroscopy was performed on a Bruker Avance-III spectrometer operating at 100.52 MHz for ^13^C equipped with a CP-MAS probe. The sample was packed in a 4 mm diameter ZrO_2_ rotor (Bruker) and then, the rotor was placed in the instrument and the MAS unit operated at 14 kHz rotational speed. The ^13^C spectrum was measured with a standard ^13^C pulse program (cp) with a number of scans of 30,000.

### 3.3. Tannin–Cellulose Interaction by Chromatographic Measurements

Interactions between tannins and bacterial cellulose were studied by adsorption experiments bringing into contact the suspended bacterial cellulose and tannin solution. The initial method presented by Phan et al. [48] was optimized for the adsorption measurements of tannins by testing different incubation times and also updated using UPLC-DAD (Acquity UPLC system, Waters Corp., Milford, MA, USA) for the measurement of the absorbance of the tannin studied. In the final method, different amounts of bacterial cellulose were incubated and shaken in 0.03 mM HT solutions or 0.06 mM PA solutions for 3 h. Different concentrations of bacterial cellulose were used: 0.003 mM, 0.004 mM, 0.005 mM, 0.008 mM and 0.016 mM, assuming the molecular weight of cellulose to be 143 kDa [78]. All adsorption experiments were performed using three replicate samples. After incubation, samples were taken from the tubes and filtered with 0.2 µm PTFE filters prior the UPLC-DAD analysis. In addition, the initial tannin solutions were filtered as such and treated as control samples (no interaction with bacterial cellulose). All samples were analysed using a UPLC BEH phenyl column (2.1 × 100 mm, 1.7 µmm Waters Corp., Wexford, Ireland). The mobile phase consisted of acetonitrile (A) and water and formic acid (99.9:0.1, *v*/*v*) (B). The elution profile was as follows: 0–0.5 min 0.1% A in B; 0.5–6.0 min 0.1–35% A in B (linear gradient), followed by column washing and stabilization. The flow rate was 0.5 mL/min and the injection volume was 5 µL. The data were processed with MassLynx software (version 4.2 SCN 982, Waters Corp., Milford, MA, USA) and the absorbance at 280 nm was used for the integration of peak areas (example chromatograms in Figure S1). The amount of tannin adsorbed to cellulose was calculated by subtracting the amount of tannin remaining in the interaction solution from the amount of tannin in the control solution (no interaction with the cellulose) and expressed as percentages.

## 4. Conclusions

The interactions and adsorption behaviour of individual HTs and well-characterized PA fractions with bacterial cellulose were studied by simple adsorption measurements using UPLC-DAD. Bacterial cellulose is an excellent model polysaccharide as it is structurally identical to plant-derived cellulose and has a high purity and crystallinity. In addition, it can be easily prepared from *nata de coco* without any cultivation or special knowledge in microbiology. The more time consuming step is the preparation and characterization of the model tannins. However, it is important that the tannins used are well characterized as the interactions are affected both by the tannin and polysaccharide structures in addition to the environmental conditions and factors. The presented method, which utilizes bacterial cellulose and adsorption measurements, is straightforward and fast to perform, and offers possibilities for rapid wider screenings of the affinities of different tannins. The results show that, for the interactions of HTs with bacterial cellulose, the most important structural features are the presence and number of free galloyl groups in the HT structures and the molecular flexibility. The flexibility of tannin structure is also important for the interactions between PAs and cellulose. The binding of A-type PAs to cellulose is weaker than the binding of B-type PAs. In addition, the binding of pure PDs is stronger in comparison to pure PCs, but in PC/PD mixture, the results are more complex to explain and the PD % is not directly linked to the strength of the interaction. Therefore, the impact of PC/PD ratio together with the stereochemistry of monomeric units would be interesting to study. Also, the role of interflavanoid linkages (C4 → C8 vs. C4 → C6) would be interesting to investigate. However, this is not easy as the interflavanoid linkages cannot be determined with the current methods for qualitative PA analysis, i.e., by mass spectrometry or degradation methods.

## Figures and Tables

**Table 1:** Hydrolysable tannins (HTs) with their molecular weights (MW), the number of ordinary hexahydroxydiphenoyl (#HHDP) groups in their structure, the number of free galloyl groups (#Galloyl) in the structures and specific structural features (Figure 1) or physicochemical properties justifying their selection as model HTs. In addition, the amounts of free HTs in solution (%) in comparison to control HT solution after incubation with 16 µM bacterial cellulose are shown (Figure 5). DOG refers to a linking unit where HHDP is the *O*-donating polyphenolic unit and galloyl is the acceptor, ET is ellagitannin, GG is galloylglucose, GOG is a linking unit where both the *O*-donating and *O*-accepting units are galloyls, nd represents no detected interactions and NHTP is the nonahydroxytriphenoyl group.

Model Tannin	MW (Da)	#HHDP	#Galloyl	Specific Structural Features	Amount of Unbound Tannin (%)
tellimagrandin I	786.6	1	2	free OH in C-1	65
vescalagin	934.6	1	0	acyclic, NHTP group	90
tellimagrandin II	938.7	1	3	hydrophobic ET	72
pentagalloylglucose	940.7	0	5	hydrophobic GG	25
geraniin	952.6	1	1	dehydro-HHDP group	nd
chebulagic acid	954.7	1	1	chebuloyl group	nd
punicalagin	1084.7	1	0	gallagyl group	nd
oenothein B	1569.1	0	2	macrocyclic dimer, 2 × DOG	nd
gemin A	1873.3	3	2	dimer, 1 × GOG	45

**Table 2:** Diverse proanthocyanidin (PA) fractions with their average molecular weight (MW), mean degree of polymerization (mDP) and procyanidin (PC):prodelphinidin (PD) ratio determined by multiple reaction monitoring by UPLC-MS/MS [67]. In addition, the amounts of free PAs in solution (%) in comparison to control PA solution after the incubation with 16 µM bacterial cellulose are shown (Figure 6). PC/PD mixture refers to PAs containing both PC and PD units.

Model Tannin	MW (Da)	mDP	PC:PD	Amount of Unbound Tannin (%)
B-type PDs	3174.3	10.4	1:99	68
B-type PCs	1530.7	5.3	99:1	81
A-type PDs	3332.6	11.0	7:93	89
A-type PCs	2993.1	10.4	99:1	92
higher PDs *	4723.0	15.4	1:99	74
galloylated PC/PD mixture	1663.1	4.6	72:28	79
PD-rich PC/PD mixture	2888.0	9.6	21:79	88
PC-rich PC/PD mixture	1810.5	6.2	77:23	85

* The fraction contains both A- and B-type PDs and minor impurities, as can be seen in Figure 3E, for example, the ions at *m*/*z* 301 and 463.

## Data Availability

The data presented in this study are available on request from the corresponding author.

## Supplementary Materials

The following supporting information can be downloaded at https://www.mdpi.com/article/10.3390/molecules28145370/s1: Figure S1. Examples of the UPLC-DAD chromatograms (at 280 nm) of hydrolysable tannins and proanthocyanidins before incubation (control solution): (A) pentagalloylglucose and (B) A-type prodelphinidins, and after incubation with 4 µM bacterial cellulose (free tannins in solution): (C) pentagalloylglucose and (D) A-type prodelphinidins; Table S1. Twenty main ions with their relative intensities and identities present in the mass spectrum of B-type prodelphinidins (PDs) presented in Figure 3 in the main text; Table S2. Twenty main ions with their relative intensities and identities present in the mass spectrum of B-type procyanidins (PCs) presented in Figure 3 in the main text; Table S3. Twenty main ions with their relative intensities and identities present in the mass spectrum of A-type prodelphinidin (PDs) presented in Figure 3 in the main text; Table S4. Twenty main ions with their relative intensities and identities present in the mass spectrum of A-type procyanidins (PCs) presented in Figure 3 in the main text; Table S5. Twenty main ions with their relative intensities and identities present in the mass spectrum of prodelphinidins (PDs) with a higher mean degree of polymerization presented in Figure 3 in the main text; Table S6. Twenty main ions with their relative intensities and identities present in the mass spectrum of galloylated proanthocyanidins containing both procyanidin (PC) and prodelphinidin (PD) units presented in Figure 3 in the main text; Table S7. Twenty main ions with their relative intensities and identities present in the mass spectrum of prodelphinidin (PD) rich mixture containing both procyanidin (PC) and prodelphinidin (PD) units presented in Figure 3 in the main text; Table S8. Twenty main ions with their relative intensities and identities present in the mass spectrum of procyanidin (PC)-rich mixture containing both procyanidin (PC) and prodelphinidin (PD) units presented in Figure 3 in the main text.

## Sample Availability

Samples of the model tannins or bacterial cellulose are not available from the authors.
