# Supplementary material for: Affinity of Tannins to Cellulose: A Chromatographic Tool for Revealing Structure-Activity Patterns
Source: Molecules. 2023 Jul 13;28(14):5370. doi: 10.3390/molecules28145370 (PMC10384774; doi:10.3390/molecules28145370)
Supplement: Supplementary file 1 [file molecules-28-05370-s001.zip › molecules-2454234-supplementary.pdf]

molecules

Supplementary material

Affinity of Tannins to Cellulose: A Chromatographic Tool for Revealing Structure–Activity Patterns

Essi Suominen [1], Santeri Savila [1], Mimosa Sillanpää [1], Pia Damlin [2] and Maarit Karonen [1,*]

[1] Natural Chemistry Research Group, Department of Chemistry, University of Turku, FI-20014 Turku, Finland; essi.m.suominen@gmail.com (E.S,), savila.santeri@gmail.com (S.S.), mamsil@utu.fi (M.S.)
[2] Materials Chemistry Research Group, Department of Chemistry, University of Turku, FI-20014 Turku, Finland; pia.damlin@utu.fi
* Correspondence: maarit.karonen@utu.fi; Tel.: +358-29-450-3179

Table of Contents

Figure S1. Examples of the UPLC-DAD chromatograms (at 280 nm) of hydrolysable tannins and proanthocyanidins before incubation (control solution): (A) pentagalloylglucose and (B) A-type prodelphinidins, and after incubation with 4 μM bacterial cellulose (free tannins in solution): (C) pentagalloylglucose and (D) A-type prodelphinidins. 2

Table S1. Twenty main ions with their relative intensities and identities present in the mass spectrum of B-type prodelphinidins (PDs) presented in Figure 3 in the main text. 3

Table S2. Twenty main ions with their relative intensities and identities present in the mass spec-trum of B-type procyanidins (PCs) presented in Figure 3 in the main text. 4

Table S3. Twenty main ions with their relative intensities and identities present in the mass spectrum of A-type prodelphinidin (PDs) presented in Figure 3 in the main text. 5

Table S4. Twenty main ions with their relative intensities and identities present in the mass spectrum of A-type procyanidins (PCs) presented in Figure 3 in the main text. 6

Table S5. Twenty main ions with their relative intensities and identities present in the mass spectrum of prodelphinidins (PDs) with a higher mean degree of polymerization presented in Figure 3 in the main text. 7

Table S6. Twenty main ions with their relative intensities and identities present in the mass spectrum of galloylated proanthocyanidins containing both procyanidin (PC) and prodelphinidin (PD) units presented in Figure 3 in the main text. 8

Table S7. Twenty main ions with their relative intensities and identities present in the mass spectrum of prodelphinidin (PD) rich mixture containing both procyanidin (PC) and prodelphinidin (PD) units presented in Figure 3 in the main text. 9

Table S8. Twenty main ions with their relative intensities and identities present in the mass spectrum of procyanidin (PC) rich mixture containing both procyanidin (PC) and prodelphinidin (PD) units presented in Figure 3 in the main text. 10

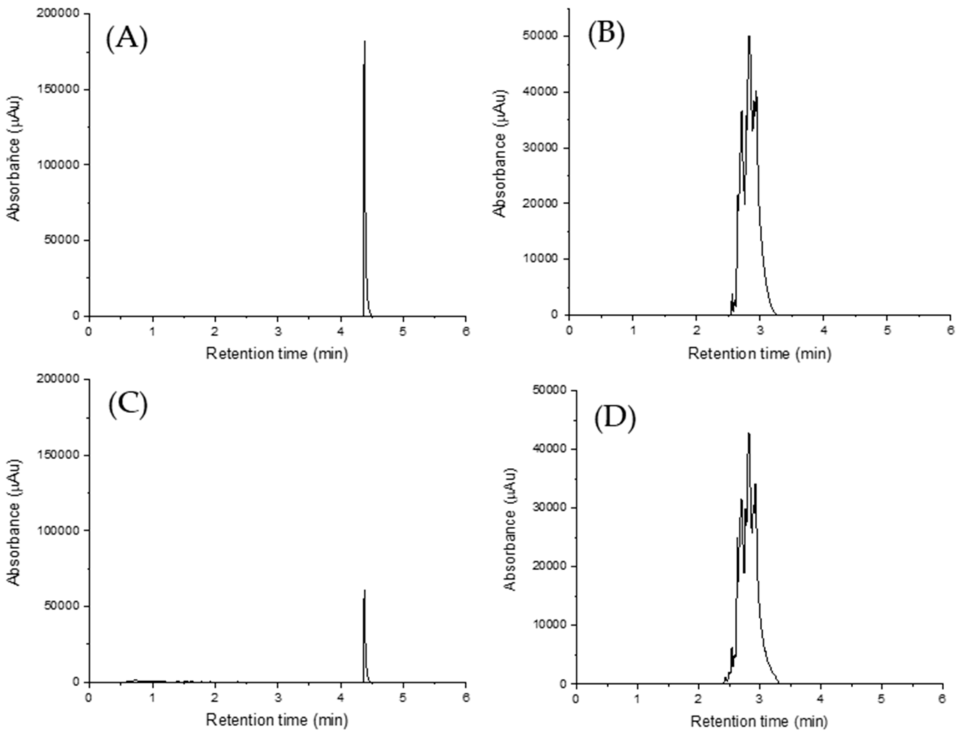

Figure S1. Examples of the UPLC-DAD chromatograms (at 280 nm) of hydrolysable tannins and proanthocyanidins before incubation (control solution): (A) pentagalloylglucose and (B) A-type prodelphinidins, and after incubation with 4 μM bacterial cellulose (free tannins in solution): (C) pentagalloylglucose and (D) A-type prodelphinidins.

Table S1. Twenty main ions with their relative intensities and identities present in the mass spectrum of B-type prodelphinidins (PDs) presented in Figure 3 in the main text.

m/z	**Relative intensity**	**Identity***
303.05	11	QM/ox
305.07	27	QM, PD monomer
423.07	18	RDA fragment
441.08	12	RDA fragment
607.11	15	QM/ox
609.12	28	PD-PD dimer
610.13	9	isotopic peak
727.13	9	RDA fragment
745.14	15	RDA fragment
911.17	12	QM/ox
912.17	8	isotopic peak
913.18	100	PD-PD-PD trimer
914.19	48	isotopic peak
915.19	16	isotopic peak
1215.23	8	QM/ox
1217.24	48	PD-PD-PD-PD tetramer
1218.24	30	isotopic peak
1219.25	12	isotopic peak
1521.30	19	PD-PD-PD-PD-PD pentamer
1522.30	15	isotopic peak

* QM refers to quinone-methide cleavage of PDs, RDA to retro-Diels-Alder fragmentation, ox to oxidation of PDs or formation of quinones during the mass spectrometric analyses [1]. Since this is a total mass spectrum, individual peaks may originate from a smaller oligomer or be fragments of a higher oligomer.

Table S2. Twenty main ions with their relative intensities and identities present in the mass spectrum of B-type procyanidins (PCs) presented in Figure 3 in the main text.

m/*z*	Relative intensity	Identity*
287.06	19	QM/ox
289.07	39	QM, PC monomer
407.08	14	RDA fragment
425.09	13	RDA fragment
575.12	33	QM/ox
577.13	34	PC-PC dimer
713.15	13	RDA fragment
859.15	14	unknown
863.18	36	QM/ox
864.19	19	isotopic peak
865.20	100	PC-PC-PC trimer
866.20	48	isotopic peak
867.20	15	isotopic peak
1151.25	23	QM/ox
1152.25	16	isotopic peak
1153.26	67	PC-PC-PC-PC tetramer
1154.27	42	isotopic peak
1155.27	17	isotopic peak
1441.32	23	PC-PC-PC-PC-PC pentamer
1442.33	17	isotopic peak

* QM refers to quinone-methide cleavage of PCs, RDA to retro-Diels-Alder fragmentation, ox to oxidation of PCs or formation of quinones during the mass spectrometric analyses [1]. Since this is a total mass spectrum, individual peaks may originate from a smaller oligomer or be fragments of a higher oligomer.

Table S3. Twenty main ions with their relative intensities and identities present in the mass spectrum of A-type prodelphinidin (PDs) presented in Figure 3 in the main text.

m/*z*	**Relative intensity**	**Identity***
303.05	22	QM/ox
305.07	57	QM, PD monomer
423.07	17	RDA fragment
427.07	16	HRF fragment
607.11	23	A-type PD-PD dimer
609.12	20	B-type PD-PD dimer
743.13	20	RDA fragment
895.17	16	A-type PC-PD-PD trimer**
911.17	83	A-type PD-PD-PD trimer
912.17	44	isotopic peak
913.18	59	isotopic peak,<br>B-type PD-PD-PD trimer
914.19	26	isotopic peak
1199.23	100	A-type PC-PD-PD-PD tetramer**
1200.23	65	isotopic peak
1201.24	37	isotopic peak,<br>B-type PC-PD-PD-PD tetramer**
1215.22	96	A-type PD-PD-PD-PD tetramer
1216.23	67	isotopic peak
1217.23	36	isotopic peak,<br>B-type PD-PD-PD-PD tetramer
1503.29	17	A-type PC-PD-PD-PD-PD pentamer**
1521.30	19	B-type PD-PD-PD-PD-PD pentamer

* QM refers to quinone-methide cleavage of PDs, RDA to retro-Diels-Alder fragmentation, ox to oxidation of PDs or formation of quinones during the mass spectrometric analyses [1]. Since this is a total mass spectrum, individual peaks may originate from a smaller oligomer or be fragments of a higher oligomer.

** the marking shows the number of PC and PD units but the sequential order is not precisely determined. There can exist several different isomers.

Table S4. Twenty main ions with their relative intensities and identities present in the mass spectrum of A-type procyanidins (PCs) presented in Figure 3 in the main text.

m/z	Relative intensity	Identity*
287.06	36	QM/ox
289.07	28	QM, PC monomer
411.07	21	HRF fragment
449.09	17	HRF fragment
573.10	29	A-type PC-PC dimer**
575.12	67	A-type PC-PC dimer
576.12	22	isotopic peak
737.15	20	HRF fragment
861.17	100	A-type PC-PC-PC trimer**
862.17	52	isotopic peak
863.18	60	A-type PC-PC-PC trimer
864.19	25	isotopic peak
1025.21	21	HRF fragment
1149.23	36	A-type PC-PC-PC-PC tetramer**
1150.23	26	isotopic peak
1151.24	32	A-type PC-PC-PC-PC tetramer
1152.25	17	isotopic peak
1437.29	25	A-type PC-PC-PC-PC-PC pentamer**
1438.30	19	isotopic peak
1439.30	17	A-type PC-PC-PC-PC-PC pentamer

* QM refers to quinone-methide cleavage of PCs, HRF to heterocyclic ring fission, RDA to retro-Diels-Alder fragmentation, ox to oxidation of PCs or formation of quinones during the mass spectrometric analyses [1]. Since this is a total mass spectrum, individual peaks may originate from a smaller oligomer or be fragments of a higher oligomer.

** having two A-type linkages

Table S5. Twenty main ions with their relative intensities and identities present in the mass spectrum of prodelphinidins (PDs) with a higher mean degree of polymerization presented in Figure 3 in the main text.

m/z	**Relative intensity**	**Identity***
167.04	11	other phenolic compound**
177.02	8	other phenolic compound**
238.89	8	unknown
261.04	8	other phenolic compound**
301.04	100	other phenolic compound**
302.04	16	isotopic peak
303.05	17	QM/ox
305.07	41	QM, PD monomer
319.05	11	other phenolic compound**
423.07	9	RDA fragment
463.09	49	other phenolic compound**
464.09	11	isotopic peak
607.11	21	A-type PD-PD dimer
608.11	7	isotopic peak
609.13	20	B-type PD-PD dimer
886.37	13	unknown
887.37	7	isotopic peak
911.17	11	A-type PD-PD-PD trimer
913.18	10	B-type PD-PD-PD trimer
1190.42	7	unknown

* QM refers to quinone-methide cleavage of PDs, RDA to retro-Diels-Alder fragmentation, ox to oxidation of PDs or formation of quinones during the mass spectrometric analyses [1]. Since this is a total mass spectrum, individual peaks may originate from a smaller oligomer or be fragments of a higher oligomer.

** Note that the efficiency of ionization decreases when the degree of polymerization of proanthocyanidins increases and therefore, the ionization of other phenolic compounds is more intensive [1].

Table S6. Twenty main ions with their relative intensities and identities present in the mass spectrum of galloylated proanthocyanidins containing both procyanidin (PC) and prodelphinidin (PD) units presented in Figure 3 in the main text.

m/*z*	Relative intensity	Identity*
169.01	45	gallic acid fragment
287.06	18	QM/ox
289.07	26	QM, PC monomer
303.05	24	QM/ox
407.08	28	RDA fragment
441.08	21	QM, galloylated PC monomer
457.08	25	QM, galloylated PD monomer
575.12	19	QM/ox
577.14	26	PC-PC dimer
729.15	100	galloylated PC-PC dimer
730.15	41	isotopic peak
745.14	54	galloylated PC-PD dimer**
746.14	22	isotopic peak
897.15	20	galloylated PC-PD dimer***
1017.21	30	galloylated PC-PC-PC trimer
1018.21	17	isotopic peak
1033.20	19	galloylated PC-PC-PD trimer**
1169.22	34	galloylated PC-PC-PC trimer***
1170.22	21	isotopic peak
1185.22	18	galloylated PC-PC-PD trimer**,***

* QM refers to quinone-methide cleavage of galloylated proanthocyanidins, RDA to retro-Diels-Alder fragmentation, ox to oxidation or formation of quinones during the mass spectrometric analyses [1]. Since this is a total mass spectrum, individual peaks may originate from a smaller oligomer or be fragments of a higher oligomer.

** the marking shows the number of PC and PD units but the sequential order is not precisely determined. There can exist several different isomers.

*** contains two galloyl groups.

Table S7. Twenty main ions with their relative intensities and identities present in the mass spectrum of prodelphinidin (PD) rich mixture containing both procyanidin (PC) and prodelphinidin (PD) units presented in Figure 3 in the main text.

m/*z*	**Relative intensity**	**Identity***
289.07	33	QM, PC monomer
303.05	22	QM/ox
305.07	51	QM, PD monomer
423.07	38	RDA fragment
425.09	21	RDA fragment
441.08	19	RDA fragment
591.12	22	QM/ox
593.13	55	PC-PD dimer**
607.11	21	QM/ox
609.13	25	PD-PD dimer
881.19	67	PC-PC-PD trimer**
882.20	33	isotopic peak
897.19	100	PC-PD-PD trimer**
898.19	48	isotopic peak
913.18	47	PD-PD-PD trimer
914.19	23	isotopic peak
1185.25	34	PC-PC-PD-PD tetramer**
1186.26	22	isotopic peak
1201.25	39	PC-PD-PD-PD tetramer**
1202.25	24	isotopic peak

* QM refers to quinone-methide cleavage, RDA to retro-Diels-Alder fragmentation, ox to oxidation or formation of quinones during the mass spectrometric analyses [1]. Since this is a total mass spectrum, individual peaks may originate from a smaller oligomer or be fragments of a higher oligomer.

** the marking shows the number of PC and PD units but the sequential order is not precisely determined. There can exist several different isomers.

Table S8. Twenty main ions with their relative intensities and identities present in the mass spectrum of procyanidin (PC) rich mixture containing both procyanidin (PC) and prodelphinidin (PD) units presented in Figure 3 in the main text.

m/*z*	**Relative intensity**	**Identity***
287.06	18	QM/ox
289.07	48	QM, PC monomer
407.08	25	RDA fragment
425.09	23	RDA fragment
575.12	28	QM/ox
577.14	41	PC-PC dimer
593.13	19	PC-PD dimer**
713.15	18	RDA fragment
865.20	100	PC-PC-PC trimer
866.20	49	isotopic peak
867.21	16	isotopic peak
879.18	17	QM/ox
881.19	64	PC-PC-PD trimer**
882.20	31	isotopic peak
897.19	28	PC-PD-PD trimer**
1153.26	54	PC-PC-PC-PC tetramer
1154.27	35	isotopic peak
1155.27	15	isotopic peak
1169.26	25	PC-PC-PC-PD tetramer**
1170.26	16	isotopic peak

* QM refers to quinone-methide cleavage of PDs, RDA to retro-Diels-Alder fragmentation, ox to oxidation of PDs or formation of quinones during the mass spectrometric analyses [1]. Since this is a total mass spectrum, individual peaks may originate from a smaller oligomer or be fragments of a higher oligomer.

** the marking shows the number of PC and PD units but the sequential order is not precisely determined. There can exist several different isomers.

References

1. Karonen, M.; Imran, I. Bin; Engström, M.T.; Salminen, J.-P. Characterization of natural and alkaline-oxidized proanthocyanidins in plant extracts by ultrahigh-resolution UHPLC-MS/MS. *Molecules* **2021**, *26*, 1873.
